# Posterior versus anterior approach for contralateral C7 nerve transfer in post-stroke upper limb spastic hemiplegia: A comparative retrospective study

**DOI:** 10.1097/MD.0000000000045086

**Published:** 2025-10-03

**Authors:** Shuide Chen, Siyuan Pan, Seidu A. Richard, Zhigang Lan

**Affiliations:** a Department of Neurosurgery, West China Hospital, Sichuan University, Chengdu, Sichuan, P.R. China; b Department of Neurosurgery, West China Xiamen Hospital, Sichuan University, Xiamen, P.R. China; c Institute of Neuroscience, Third Affiliated Hospital, Zhengzhou University, Zhengzhou, China; d Department of Biochemistry and Forensic Sciences, School of Chemical and Biochemical Sciences, C. K. Tedam University of Technology and Applied Sciences (CKT-UTAS), Navrongo, Ghana.

**Keywords:** contralateral C7, hemiplegia, nerve root transfer, spasticity, stroke

## Abstract

Several surgical approaches for contralateral C7 (CC7) nerve root transfer (NRT) exist, but they come with challenges such as long nerve bridging distances, complex surgical anatomy, and prolonged recovery times. Our objective was to evaluate and compare the clinical efficacy and safety of the posterior single-incision CC7 (PSCC7) NRT approach (posterior group) with the traditional anterior CC7 (ACC7) NRT approach (anterior group) for treating post-stroke upper limb spastic hemiplegia (PULSH). In this retrospective study, we retrieved and compared clinical efficacy as well as safety of the posterior group with the traditional anterior group for treating PULSH between February 2024 and February 2025. Key outcome measures included operative time, intraoperative blood loss, postoperative complications, and functional recovery at 6-month follow-up. In all, 12 patients who underwent the posterior group and 30 patients (control group) who underwent the traditional anterior group were retrieved from the hospital records. The posterior group demonstrated significantly shorter operative times (3.2 ± 0.5 hours vs 4.5 ± 0.8 hours, *P* < .01) and less intraoperative blood loss (150 ± 4 0 mL vs 280 ± 70 mL, *P* < .01) compared to the anterior group. The posterior group achieved comparable or superior improvements in Modified Ashworth Scale scores (1.2 ± 0.3 vs 1.5 ± 0.4, *P* > .05) and Berg Balance Scale scores (42 ± 5 vs 39 ± 6, *P* > .05). The posterior group is a safe and effective treatment for PULSH. It offers the advantages of reduced surgical trauma and shorter operative time.

## 1. Introduction

Stroke remains a leading cause of long-term disability worldwide, with nearly 40% of survivors developing spastic hemiplegia.^[1,2]^ This condition is characterized by muscle hypertonia and impaired motor control which severely limits daily activities of living and imposes a significant socioeconomic burden.^[3]^ Conventional therapies such as physical rehabilitation, oral antispasmodics like baclofen and botulinum toxin injections often provide only transient symptomatic relief.^[4,5]^ They frequently fail to address the underlying challenge of restoring voluntary, functional motor control in the affected upper limb leaving a substantial population of patients with persistent debilitating impairment.^[6]^

A paradigm shift in restorative neurosurgery has emerged focusing on leveraging the brain’s inherent neuroplasticity.^[7]^ The contralateral C7 (CC7) nerve root transfer (NRT), a technique pioneered by Gu et al for brachial plexus injuries, has been adapted for central nervous system deficits.^[8]^ The procedure involves rerouting the C7 NRT from the healthy side to innervate the paralyzed limb.^[8]^ This establishes a new neural circuit enabling the uninjured cerebral hemisphere to assume control over the affected arm.^[8]^ Landmark studies such as the randomized controlled trial (RCT) by Zheng et al have provided evidence that CC7 NRT, combined with rehabilitation significantly improves motor function and reduces spasticity in patients with chronic stroke, thereby validating it as a powerful therapeutic strategy.^[9]^

In recent years, several studies have further explored the application of CC7 NRT in stroke recovery. Alawieh et al highlighted that CC7 NRT represents a new frontier for peripheral nerve surgery in stroke recovery, discussing its potential mechanisms and preliminary clinical outcomes while also noting the need for more comparative studies to optimize surgical approaches.^[10]^ Additionally, a case report by Zhang et al described the successful use of CC7 NRT in the treatment of central hemiplegia after stroke under general anesthesia, providing real-world evidence of the technique’s feasibility in individual patients.^[11]^ These studies, along with others, have contributed to the growing body of literature on CC7 NRT but also underscored the lack of head-to-head comparisons between different surgical approaches for this technique.

Historically, CC7 NRT has been performed via an anterior (ACC7) NRT approach (anterior group). This technique involves bilateral supraclavicular incisions and dissection through a complex anatomical field adjacent to vital mediastinal structures such as the trachea, esophagus, and carotid artery.^[12]^ A key limitation of the ACC7 NRT route is the long transfer distance which often necessitates the use of nerve grafts such as sural nerve to bridge the gap. This introduces 2 anastomosis sites which potentially hinders nerve regeneration. Furthermore, the procedure is associated with a notable incidence of donor-site morbidity such as transient dysphagia, neck pain, and sensory deficits in the healthy hand.^[13–15]^

To mitigate the challenges of the traditional anterior group, a posterior single-incision contralateral C7 (PSCC7) NRT approach (posterior group) was developed. This technique aims to provide a more direct and shorter pathway for nerve transfer thereby reducing surgical trauma and eliminating the need for grafts.^[15,16]^ We hypothesized that the posterior group would be safer and more efficient than the traditional anterior group while achieving comparable or superior clinical outcomes. The primary objective of this study is to directly compare the surgical efficiency, a safety profile and functional results of the posterior group versus the traditional anterior group in a cohort of patients with post-stroke upper limb spastic hemiplegia (PULSH).

## 2. Materials and methods

### 2.1. Study design and patient selection

A single-center retrospective cohort study was conducted at Huaxi Xiamen Hospital (Sichuan University) from February 2024 to February 2025. The institutional review boards of Sichuan University approved this study (Approval No. HX-2024-01). The patients and their relatives fully consented to the use of their information after dually informing them about our intention to involve them in a study. Patients’ medical records, surgical reports, and rehabilitation assessments records were retrieved from hospital records. The posterior group included all consecutive patients who underwent posterior group for PULSH at our institution between February 2024 and February 2025. Also, anterior group (control group) consisted of patients who underwent a traditional anterior group for PULSH which the same time period indication above. Key outcome measures included operative time, intraoperative blood loss, postoperative complications, and functional recovery at 6-month follow-up. Modified Ashworth Scale (MAS) for spasticity and the Berg Balance Scale (BBS) for balance were used for the assessments. Addition measures for only the PSCC7 nerve root transfer group included muscle tone reduction, balance improvement, and electrophysiological recovery.

To ensure comprehensive evaluation of baseline characteristics, the following additional assessments were recorded for each patient:

1. Stroke severity: assessed using the National Institutes of Health Stroke Scale (NIHSS) and the modified Rankin Scale at the time of stroke onset and preoperatively.
2. Stroke location: categorized as cortical which includes primary motor cortex, premotor cortex or subcortical which includes basal ganglia, internal capsule, thalamus and documented by hemisphere such as left or right based on preoperative cranial computed tomography and magnetic resonance imaging (MRI).
3. Comorbid neurological conditions: evaluation was done using hemispatial neglect such as line bisection test and cancelation tasks. Cognitive impairment was assessed using the Mini-Mental State Examination (MMSE) with scores < 24 indicating cognitive impairment and ataxia was assessed using clinical examination of limb coordination, gait, and balance.

### 2.2. Inclusion and exclusion criteria

Patients were included if they met the following criteria: chronic PULSH diagnosed more than 12 months post-stroke; failure to achieve significant functional improvement after at least 6 months of structured conservative therapy such as neurorehabilitation and botulinum toxin injections; preserved anatomical and functional integrity of the CC7 nerve root which was confirmed by cervical spine MRI and electromyography (EMG); age between 18 and 75 years. Exclusion criteria were: patients with severe cardiopulmonary disease or other absolute contraindications to general anesthesia; history of prior cervical spine surgery; severe cognitive impairment that would preclude cooperation with postoperative rehabilitation protocols.

### 2.3. Preoperative evaluations for the posterior group

**Figure 1.**
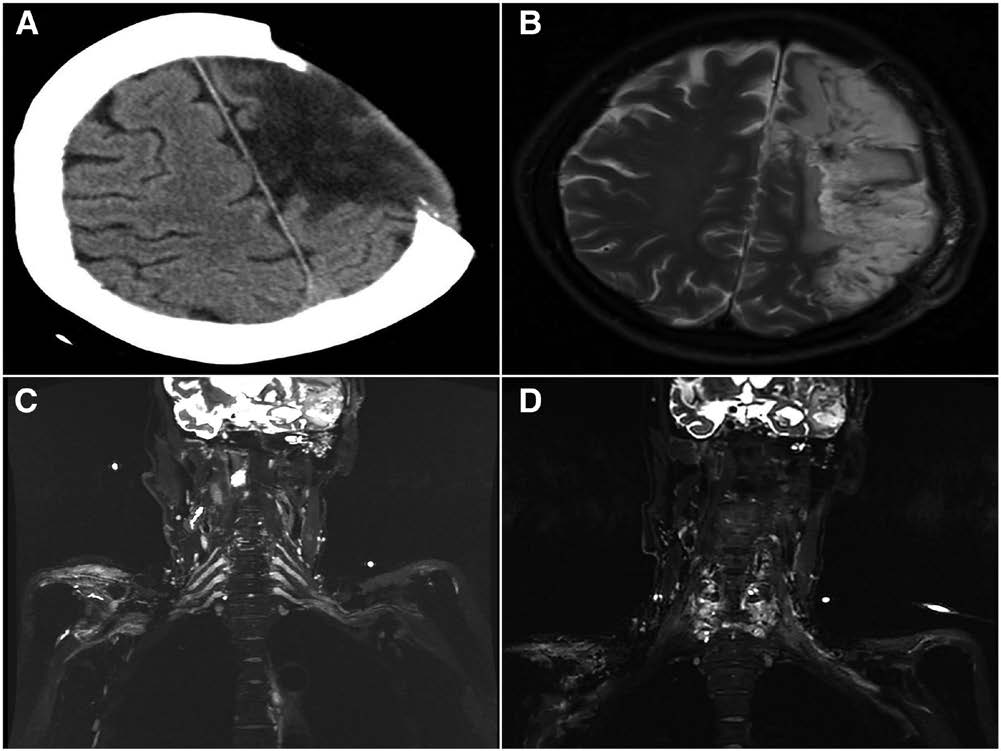
Are preoperative images of a patient who underwent PSCC7 approach. (A) Is preoperative cranial CT scan showing extensive infarction in the left hemisphere. The patient underwent decompressive craniectomy because of the large infarction. (B) Is a preoperative MRI showing significant functional area damage, notably in the motor area for the upper limb on the left side. (C and D) Preoperative MRI of the cervical spine showing the nerve roots of C4 to C7 and the surround tissues.

In all patients, initial computed tomography scan was used evaluate infarction in the hemispheres (Fig. 1A), while MRI was used to confirmed functional area damages, notably in the motor areas for the upper limbs (Fig. 1B). Also, MRI of the cervical spine was used quantify the lengths of the nerve roots of C4 to C7 (Fig. 1C and D). In all patient’s EMG was used evaluate conduction velocity and amplitude in the affected upper limb’s brachial plexus.

Additionally, transcranial magnetic stimulation (TMS) was performed to assess motor evoked potentials (MEPs) in the affected upper limb. The resting motor threshold (RMT) and MEP amplitude were recorded to evaluate the integrity of the corticospinal tract. These electrophysiological data (EMG and TMS results) were documented as part of the preoperative assessment for both groups.

### 2.4. Surgical techniques

#### 2.4.1. Surgical technique for the posterior group

**Figure 2.**
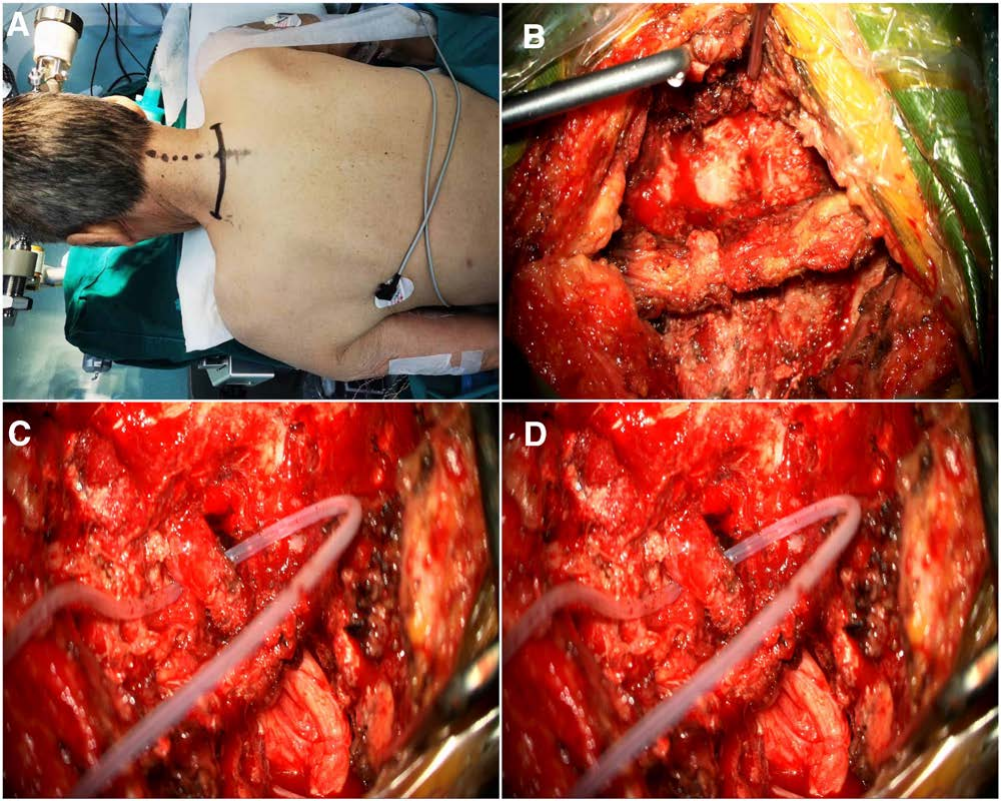
Are intraoperative images of a patient who underwent PSCC7 approach. (A) The patient in a prone positioning and the markings of a single posterior incision at the C7 level. (B) The dissection from the skin, subcutaneous tissue, and supraspinous ligament to expose the 6th and 7th cervical vertebral laminae. (C) The C7 nerve on the healthy side. (D) The anastomoses of the left C7 root to the right C7 root’s distal end with a 9–0 suture.

All patients underwent a PSCC7 nerve root transfer under general anesthesia. Patients were put in a prone position with Mayfield head fixation and 30-degree rotation to facilitate microscopic visualization. A 6 to 8 cm horizontal incision was made at the C7 to T1 level (Fig. 2A). Laminectomy of C6 to C7 was performed, preserving the ligamentum flavum to avoid spinal instability (Fig. 2B). The contralateral C7 root was dissected under neurophysiological monitoring and coopted to the ipsilateral C7 using 9–0 nylon sutures (Fig. 2C). Layers were closed with absorbable sutures (Fig. 2D), and a negative-pressure drain was placed temporarily.

#### 2.4.2. Surgical technique for the anterior group

Patients were positioned supine with the neck slightly extended. Bilateral supraclavicular incisions were made. On the donor side, dissection proceeded through the platysma and sternocleidomastoid muscle to expose the brachial plexus. The C7 root was identified and transected. A retropharyngeal or subcutaneous tunnel was created to pass the nerve to the recipient side. On the recipient side, a similar dissection exposed the brachial plexus. The recipient C7 root was prepared for anastomosis. If direct, tension-free coaptation was not possible, an interpositional nerve graft (typically a harvested sural nerve) was used to bridge the gap.

### 2.5. Postoperative rehabilitation

Postoperative rehabilitation was same for both groups. Phase 1 (0–4 weeks): cervical collar immobilization to protect the nerve graft while phase 2 (4–12 weeks): passive range-of-motion exercises and neuromuscular electrical stimulation to prevent disuse atrophy. Phase 3 (>12 weeks): task-specific training such as grasping, weight-bearing tasks were used to promote neuroplasticity.

### 2.6. Outcome measures

In primary outcome assessments, MAS scores for elbow, wrist, and finger flexors measured preoperatively and at the 6-month postoperative follow-up was used to assess spasticity while BBS scores measured preoperatively and at 6 months postoperatively was used to assess balance. In secondary outcomes assessments, operative time (in minutes) and intraoperative blood loss (in mL) were used to assess surgical efficiency while incidence of intraoperative events such as dural tear and vascular injury, postoperative complications such as wound infection and delayed healing and specific donor-site morbidities such as neck pain, sensory deficits, and dysphagia were used to asses safety and complications. Also, changes in EMG amplitude and conduction velocity were assessed at 3 months postoperatively to confirm early reinnervation.

### 2.7. Statistical analysis

Statistical analysis was performed using SPSS v27.0 (IBM Corp., Armonk). Independent *t* tests were used to compare continuous variables such as age, operative time, blood loss, and MAS/BBS scores between the 2 groups. For nonnormally distributed data, the Mann–Whitney *U* test was applied. Categorical variables such as sex, complication rates were compared using the Chi-square test or Fisher exact test as appropriate.

Subgroup analysis was conducted to compare outcomes such as operative time, blood loss, MAS, and BBS between patients with right upper limb involvement and left upper limb involvement within each surgical group. Additionally, multivariate linear regression analysis was performed to adjust for potential confounders such as age, stroke duration, preoperative NIHSS score, stroke location, and presence of cognitive impairment when comparing functional outcomes between the 2 groups. *P*-value of <.05 was considered statistically significant.

## 3. Results

### 3.1. Patient demographics and baseline characteristics

A total of 42 patients with PULSH were retrieved from the records. Twelve^[12]^ patients underwent the posterior group while 30 underwent the anterior group. The demographic and baseline clinical characteristics were well-matched between the 2 groups (Table 1). For age, the posterior group had a mean ± SD of 65 ± 4.2 years (interquartile range [IQR]: 62–68 years) and the anterior group had 63 ± 5.1 years (IQR: 60–67 years), with a *P*-value of .283. The sex distribution was 7 males and 5 females in the posterior group and 17 males and 13 females in the anterior group (*P* = .891). Regarding the affected side, 7 patients had right upper limb involvement and 5 had left in the posterior group, while 16 had right and 14 had left in the anterior group (*P* = .924). The mean ± SD for time since stroke stared was 14 ± 3.1 months (IQR: 12–16 months) in the posterior group and 15 ± 3.5 months (IQR: 13–18 months) in the anterior group (*P* = .367).

**Table 1:** Demographic and baseline characteristics of patients.

Parameter	PSCC7 NRT approach (n = 12)	ACC7 NRT approach (n = 30)	*P*-value
Age (yr, mean ± SD; IQR)	65 ± 4.2; 62–68	63 ± 5.1; 60–67	.283
Sex (male/female, n)	7/5	17/13	.891
Affected side (right/left, n)	7/5	16/14	.924
Time since stroke (mo, mean ± SD; IQR)	14 ± 3.1; 12–16	15 ± 3.5; 13–18	.367
Preoperative MAS (mean ± SD; IQR)	3.8 ± 0.4; 3.5–4.0	3.7 ± 0.5; 3.3–4.0	.582
Preoperative BBS (mean ± SD; IQR)	28 ± 6; 24–32	27 ± 7; 22–33	.693
Preoperative NIHSS (mean ± SD)	6.2 ± 2.1	5.8 ± 2.3	.568
Preoperative mRS (median [IQR])	3 [2–4]	3 [2–4]	.712
Cortical stroke (n, %)	5 (41.7%)	13 (43.3%)	.915
Left hemisphere stroke (n, %)	6 (50.0%)	15 (50.0%)	1.000
Hemispatial neglect (n, %)	1 (8.3%)	3 (10.0%)	.857
Cognitive impairment (MMSE < 24, n, %)	2 (16.7%)	5 (16.7%)	1.000
Ataxia (n, %)	0 (0%)	2 (6.7%)	.481

BBS = Berg Balance Scale (used to assess balance, with scores ranging from 0 to 56, where higher scores indicate better balance), MAS = Modified Ashworth Scale (used to assess spasticity, with scores ranging from 0 to 4, where higher scores indicate greater spasticity), MMSE = Mini-Mental State Examination (used to assess cognitive function, with scores ranging from 0 to 30, where lower scores indicate greater cognitive impairment), mRS = modified Rankin Scale (used to assess disability, with scores ranging from 0 to 6, where higher scores indicate greater disability), NIHSS = National Institutes of Health Stroke Scale (used to assess stroke severity, with scores ranging from 0 to 42, where higher scores indicate more severe stroke).

Preoperative MAS scores were 3.8 ± 0.4 (IQR: 3.5–4.0) in the posterior group and 3.7 ± 0.5 (IQR: 3.3–4.0) in the anterior group (*P* = .582). Preoperative BBS scores were 28 ± 6 (IQR: 24–32) in the posterior group and 27 ± 7 (IQR: 22–33) in the anterior group (*P* = .693). Additional baseline characteristics were also well-balanced between the 2 groups:

1. Stroke severity: preoperative NIHSS score using mean ± SD revealed 6.2 ± 2.1 for the posterior group and 5.8 ± 2.3 for the anterior group with *P* = .568. Also, preoperative modified Rankin Scale score using median [IQR] revealed 3 [2–4] for the posterior group and 3 [2–4] for the anterior group with *P* = .712.
2. Stroke location: cortical stroke evaluation revealed 5 (41.7%) for the posterior group and 13 (43.3%) for the anterior group with *P* = .915. Also, left hemisphere stroke assessment revealed 6 (50.0%) for the posterior group and 15 (50.0%) for the anterior group with *P* = 1.000.
3. Comorbid neurological conditions: hemispatial neglect assessment revealed 1 (8.3%) for the posterior group and 3 (10.0%) for the anterior group with *P* = .857. Also, cognitive impairment (MMSE < 24) evaluation revealed 2 (16.7%) for the posterior group, 5 (16.7%) for the anterior group with *P* = 1.000. Furthermore, ataxia assessments revealed 0 (0%) for the posterior group and 2 (6.7%) for the anterior group with *P* = .481.

### 3.2. Surgical and perioperative outcomes

The posterior group demonstrated significant advantages in surgical efficiency (Table 2). The mean operative time for the posterior group was 192 ± 30 minutes (3.2 hours) which was significantly shorter than the 270 ± 48 minutes (4.5 hours) for the anterior group with a statistically significant difference (*P* < .01). Similarly, intraoperative blood loss was nearly halved in the posterior group (150 ± 40 mL) compared to 280 ± 70 mL for the anterior group with a statistically significant difference (*P* < .01). Critically, no patients (0/12) in the posterior group required a nerve graft for anastomosis whereas 8 of 30 patients (26.7%) in the anterior group required a sural nerve graft due to insufficient nerve length for a tension-free repair.

**Table 2:** Comparison of surgical and perioperative outcomes.

Parameter	PSCC7 NRT approach (n = 12)	ACC7 NRT approach (n = 30)	*P*-value
Operative time (min, mean ± SD)	192 ± 30	270 ± 48	<.01
Intraoperative blood loss (mL, mean ± SD)	150 ± 40	280 ± 70	<.01
Nerve graft required (n, %)	0 (0%)	8 (26.7%)	–

*Note*: The row for “nerve graft required” is descriptive, as the PSCC7 group had 0 cases, making statistical comparison (*P*-value) inappropriate.

### 3.3. Clinical and functional outcomes at follow-up

At the 6-month follow-up, both groups showed significant functional improvements compared to their preoperative baselines (Table 3). In terms of spasticity reduction, the mean MAS score in the posterior group decreased to 1.2 ± 0.3, while the anterior group score decreased to 1.5 ± 0.4. Although the posterior group showed a trend towards greater reduction, the difference between the groups was not statistically significant (*P* = .087). For balance, the mean BBS score in the posterior group improved to 42 ± 5 compared to 39 ± 6 in the anterior group. This difference was also not statistically significant (*P* = .124) indicating comparable efficacy in functional recovery at this time point.

**Table 3:** Comparison of functional outcomes at 6-month follow-up.

Parameter	PSCC7 NRT approach (n = 12)	ACC7 NRT approach (n = 30)	*P*-value
Postoperative MAS (mean ± SD)	1.2 ± 0.3	1.5 ± 0.4	.087
Postoperative BBS (mean ± SD)	42 ± 5	39 ± 6	.124
Change in EMG conduction velocity (m/s, mean ± SD)	13.1 ± 4.2	11.7 ± 3.9	.316
Change in EMG amplitude (mV, mean ± SD)	1.3 ± 0.4	1.2 ± 0.3	.458
Change in TMS RMT (%) (mean ± SD)	-9.2 ± 3.1	-7.7 ± 2.8	.225
Change in TMS MEP amplitude (mV, mean ± SD)	0.9 ± 0.2	0.8 ± 0.2	.387

BBS = Berg Balance Scale (assesses balance, 0–56 scale), EMG = electromyography (assesses nerve conduction), MAS = Modified Ashworth Scale (assesses spasticity, 0–4 scale), MEP = motor evoked potential (higher amplitudes indicate better corticospinal tract function), RMT = resting motor threshold (lower values indicate better corticospinal tract excitability), TMS = transcranial magnetic stimulation.

Subgroup analysis based on the affected upper limb side revealed no significant differences in outcomes within each group:

1. PSCC7 group: right upper limb involvement (n = 7) revealed a postoperative MAS of 1.1 ± 0.3 and BBS of 43 ± 4. Also, left upper limb involvement (n = 5) revealed postoperative MAS of 1.3 ± 0.2 and BBS of 41 ± 6. A significant difference with *P* > .05 was detected for both.
2. ACC7 group: right upper limb involvement (n = 16) revealed postoperative MAS of 1.4 ± 0.4 and BBS of 40 ± 5. Also, left upper limb involvement (n = 14) revealed postoperative MAS of 1.6 ± 0.3 and BBS of 38 ± 7. A significant difference with *P* > .05 was detected for both.

Multivariate linear regression analysis, adjusting for age, stroke duration, preoperative NIHSS score, stroke location, and cognitive impairment, confirmed that the difference in postoperative MAS (β = -0.28, 95% CI: -0.61 to 0.05, *P* = .092) and BBS (β = 2.76, 95% CI: -0.58 to 6.10, *P* = .103) between the 2 groups remained nonsignificant.

Electrophysiological assessments at 3 months confirmed early signs of reinnervation in both groups. In the posterior group, EMG conduction velocity increased from a preoperative mean ± SD of 32.1 ± 5.3 m/s to 45.2 ± 6.1 m/s (*P* < .01), and amplitude increased from 0.8 ± 0.3 mV to 2.1 ± 0.5 mV (*P* < .01). In the anterior group, conduction velocity increased from 31.8 ± 5.5 m/s to 43.5 ± 5.8 m/s (*P* < .01), and amplitude increased from 0.7 ± 0.2 mV to 1.9 ± 0.4 mV (*P* < .01). There were no significant differences in the magnitude of change in EMG parameters between the 2 groups (*P* > .05). For TMS, the posterior group showed a decrease in RMT from 58.3 ± 7.2% to 49.1 ± 6.5% (*P* < .01) and an increase in MEP amplitude from 0.3 ± 0.1 mV to 1.2 ± 0.3 mV (*P* < .01) at 6 months. The anterior group had similar changes with a decreased RMT from 57.9 ± 7.5% to 50.2 ± 6.8% (*P* < .01) and an increased MEP amplitude from 0.2 ± 0.1 mV to 1.0 ± 0.2 mV (*P* < .01), with no significant between-group differences (*P* > .05).

### 3.4. Complications and donor-site morbidity

The safety profile of the posterior group was markedly superior (Table 4). In the posterior group, only 1 (8.3%) patient experienced a complication such as delayed wound healing which was managed conservatively. Importantly, there were no (0%) instances of donor-site morbidity. In contrast, the anterior group had a significantly higher rate of complications. Six (20%) patients reported transient dysphagia or a foreign body sensation while swallowing and 9 (30%) patients experienced transient sensory changes (paresthesia) or mild weakness in the donor hand. The overall rate of donor-site morbidity was significantly lower in the posterior group (0% vs 30%, *P* < .05).

**Table 4:** Comparison of complications and donor-site morbidity.

Complication	PSCC7 NRT approach (n = 12)	ACC7 NRT approach (n = 30)	Odds ratio (OR) [95% CI]	*P*-value
Delayed wound healing (n %)	1 (8.3%)	0 (0%)	–	.28
Transient dysphagia (n %)	0 (0%)	6 (20%)	0.00 [0.00, 1.31]	.11
Transient donor-site paresthesia (n %)	0 (0%)	9 (30%)	0.00 [0.00, 0.86]	.04
Any donor-site morbidity (n%)	0 (0%)	9 (30%)	0.00 [0.0, 0.86]	.04

## 4. Discussion

This study provides compelling evidence that the posterior group is a superior surgical alternative to the traditional anterior group for treating PULSH. Our principal finding is that while both techniques achieve comparable and significant reductions in spasticity and improvements in balance at 6 months, the posterior group is markedly more efficient and safer. It significantly reduces operative time, minimizes blood loss and virtually eliminates the donor-site morbidity that has long been a concern with anterior group.^[13–15]^

The superiority of the posterior group can be attributed to fundamental anatomical and technical advantages. The posterior group through the epidural space offers the most direct anatomical path between the contralateral and ipsilateral C7 nerve roots. This shortens the required nerve transfer distance as supported by cadaveric studies^[17–19]^ and obviates the need for interpositional nerve grafts. A single tension-free anastomosis site is believed to promote more robust and rapid axonal regeneration.^[20]^

Also, the anterior group necessitates navigating a crowded anatomical corridor risking injury to the carotid sheath, trachea, esophagus, and recurrent laryngeal nerve.^[21,22]^ In contrast, the posterior dissection is more straightforward proceeding through muscle and bone while avoiding these vital anterior neck structures. This directly translates to lower intraoperative blood loss and a near-absence of procedure-related complications like dysphagia.^[14]^ Furthermore, from a practical standpoint, a single posterior incision is more ergonomic for the surgical team and offers a better cosmetic result as the scar is easily concealed by clothing or hair which can alleviate potential psychological distress for the patient.

In a pioneering move in 1986, Gu et al reported the successful use of CC7 NRT to treat brachial plexus root avulsion injury.^[8,17]^ It was discovered that the healthy hemisphere could independently control both the paralyzed and the healthy upper limbs, leading to a novel treatment strategy for PULSH.^[23–27]^ This strategy, known as “interchanging left and right nerves,” allows 1 cerebral hemisphere to control both hands, offering a unique model for studying brain plasticity.

In a 5 years single-center trial conducted by Zheng et al involving patients with unilateral arm paralysis as a result of chronic cerebral injury, transfer of the C7 nerve from the healthy arm to the paralyzed arm correlated with a better enhancement in function as well as decrease of spasticity compared to rehabilitation alone over a 12 months period.^[9]^ Notable, the study observed the association of physiological connectivity between the ipsilateral cerebral hemisphere and the paralyzed arm.^[9]^ Also, MAS, an assessment parameter for spasticity, congruently begun to reduce in the paralyzed elbows as well as wrists as soon as after surgery.^[9]^

Zheng et al also revealed that the second phase of recovery was depicted with enhancements in muscle power as well as motor function, which were most obvious at about 10 months post-surgery, perhaps signifying the time sequence of the regeneration of nerve fibers in the gap between the distal end of the transferred nerve, and more distally, on the side of the paralyzed arm.^[9]^ However, the majority of clinical enhancements corrected with physiological evidence of connectivity between the hemisphere on the side of the transferred nerve and the paralyzed arm.^[9]^

In the posterior group, our preoperative assessment using EMG to assess the nerve conduction velocity of the brachial plexus in both upper limbs, revealed a significant decrease in conduction velocity and amplitude on the affected sides. Transcranial magnetic stimulation was also used to evaluate motor evoked potentials. MRI of the brachial plexus helped us to preoperatively assess the C7 nerve root’s length, which is crucial for determining the feasibility of anastomosis without tension and the need for nerve bridging.

Guan et al performed a CC7 NRT via the posterior vertebral approach combined with selective posterior rhizotomy of the affected cervical nerve in the treatment of central upper limb spastic paralysis with a successful outcome.^[28]^ Postoperatively, their patient experienced an immediate reduction in spasticity, with significant improvements in muscle strength and motor function as detected by score such as MAS tone score, Brunstrom staging, FMA of right upper limb, WOLF motor function test, Modified Barthel index, Action Research Arm Test, and Visual Analog Scale 6 months after surgery months post-surgery. The complication rates observed in our anterior group included 20% dysphagia and 30% donor-site sensory changes which align with historical reports^[29–31]^ reinforcing the deduction that the posterior group offers a significant safety advantage.

It is important to acknowledge the role of stroke location and hemisphere in motor recovery, as highlighted by Dodd et al.^[32]^ Their study emphasized that the contralesional and ipsilesional hemispheres contribute differently to stroke recovery, with lesion location influencing neuroplasticity potential.^[32]^ In our study, we documented stroke location such as cortical versus subcortical and hemisphere such as left versus right and found no significant differences between the 2 groups, suggesting that these factors did not bias our comparison of surgical approaches. Additionally, subgroup analysis based on the affected upper limb side such as proxy for stroke hemisphere showed no significant differences in outcomes within each group, further supporting the robustness of our findings.

## 5. Limitations and future directions

This study is not without limitations. Its retrospective design carries an inherent risk of selection bias, as patients were not randomly assigned to the 2 surgical groups. The sample size, particularly in the posterior group (n = 12), is small, which may limit the statistical power to detect small but clinically meaningful differences in outcomes. The experience is from a single center, which may limit the generalizability of our findings to other institutions with different surgical expertise and patient populations.

Furthermore, the 6-month follow-up period is sufficient to assess early safety and efficacy but may not capture the full arc of motor recovery which can continue for over a year, as noted in previous study by Zheng et al.^[9]^ We also acknowledge the absence of long-term data on donor-site morbidity and functional outcomes beyond 6 months, which are important for evaluating the durability of the posterior group.

Another limitation is the lack of detailed assessment of some potential confounders, such as the intensity and duration of preoperative rehabilitation, which may have influenced postoperative outcomes. Additionally, while we evaluated cognitive impairment using the MMSE, more comprehensive neurocognitive assessments such as attention, executive function could provide a better understanding of how cognitive factors affect rehabilitation and functional recovery after nerve transfer.

The promising results in this study warrant further investigation. The gold standard would be a multicenter prospective RCT with a larger patient cohort and long-term follow-up of 2 to 5 years. RCT would definitively establish the advantages of the posterior group and could explore more nuanced questions such as identifying ideal patient candidates based on factors like stroke location, severity, and duration and optimizing postoperative rehabilitation protocols to maximize functional gains. Future studies should also include patient-reported outcomes such as quality of life, satisfaction with surgery to provide a more holistic assessment of the benefits of the posterior group.

## 6. Conclusions

The posterior group represents a promising technical advancement in the surgical management of PULSH. This technique provides a safer and more efficient alternative to the traditional anterior group without compromising clinical efficacy by minimizing surgical trauma and optimizing the nerve transfer pathway. It effectively addresses the key limitations of the anterior group, such as long operative time, significant blood loss, and high donor-site morbidity.

While the results of this study are encouraging, it is important to interpret them with caution given the retrospective design, small sample size, and short follow-up period. The PSCC7 approach should not be considered a “paradigm shift” at this stage but rather a valuable new option that requires further validation. Future prospective, multicenter RCTs with larger sample sizes and longer follow-up are needed to confirm the long-term safety and efficacy of the posterior group and to determine its role in the broader landscape of stroke rehabilitation.

Nonetheless, our findings suggest that the posterior group has the potential to improve the care of patients with PULSH who have failed conservative therapy, offering new hope for functional recovery to stroke survivors.

## Author contributions

**Conceptualization:** Shuide Chen, Siyuan Pan, Seidu A. Richard, Zhigang Lan.

**Data curation:** Shuide Chen, Siyuan Pan, Seidu A. Richard, Zhigang Lan.

**Formal analysis:** Shuide Chen, Siyuan Pan, Seidu A. Richard, Zhigang Lan.

**Investigation:** Shuide Chen, Siyuan Pan, Seidu A. Richard, Zhigang Lan.

**Methodology:** Shuide Chen, Siyuan Pan, Seidu A. Richard, Zhigang Lan.

**Software:** Shuide Chen, Siyuan Pan, Seidu A. Richard, Zhigang Lan.

**Writing – review & editing:** Shuide Chen, Siyuan Pan, Seidu A. Richard, Zhigang Lan.

**Writing – original draft:** Seidu A. Richard, Zhigang Lan.

**Funding acquisition:** Zhigang Lan.

**Resources:** Zhigang Lan.
